# Future Caregiving Scenarios: Effects of Technology Innovation and Adoption

**DOI:** 10.1093/geroni/igaa057.3038

**Published:** 2020-12-16

**Authors:** John Rudnik, Lisa D’Ambrosio, Chaiwoo Lee, Joseph Coughlin

**Affiliations:** MIT, Cambridge, Massachusetts, United States

## Abstract

Based on the expert panel’s response to the first round of the study, four scenarios that describe possible futures for the intersection of caregiving and technology were created. These scenarios differ along two dimensions – focus of technology deployment and adoption promotion – along which stakeholders’ decisions make a crucial difference for outcomes. These four scenarios describing possibilities for the year 2030 will be illustrated during this presentation, along with results from the second round of the study, which include the expert panel’s evaluations of their likelihood and impacts on caregiving.

